# Exploring the Role of Animal Technologists in Implementing the 3Rs: An Ethnographic Investigation of the UK University Sector

**DOI:** 10.1177/0162243917718066

**Published:** 2017-08-10

**Authors:** Beth Greenhough, Emma Roe

**Affiliations:** 1 School of Geography and the Environment, Oxford University Centre for the Environment, University of Oxford, Oxford, UK; 2 Geography and Environment, University of Southampton, Southampton, UK

**Keywords:** ethics, engagement, intervention, expertise, politics, power, governance

## Abstract

The biomedical industry relies on the skills of animal technologists (ATs) to put laboratory animal welfare into practice. This is the first study to explore how this is achieved in relation to their participation in implementing refinement and reduction, two of the three key guiding ethical principles––the “3Rs”––of what is deemed to be humane animal experimentation. The interpretative approach contributes to emerging work within the social sciences and humanities exploring care and ethics in practice. Based on qualitative analysis of participant observation within animal research facilities in UK universities, in-depth interviews with ATs, facility managers, and other stakeholders, and analysis of regulatory guidelines, we draw a contrast between the minimum required of ATs by law and how their care work not only meets but often exceeds these requirements. We outline how ATs constitute a key source of innovation and insight into the refinement of animal care and the reduction of animal use, hitherto not formally acknowledged. Exploring AT care work as an example of ethics in practice makes an original contribution to broader debates within health care and animal welfare about how technology, regulation, and behavior can foster and sustain a “culture of care”.

## Introduction

Ethical review procedures for animal experimentation rarely hear the voice of the (junior) animal technologists (ATs)^1^, who carry out the day-to-day care for research animals. They are involved in the breeding and preparation of animals for use in research and are sometimes called upon to carry out licensed experimental procedures such as tail injections or gavaging (administration of drug or similar directly into the stomach through a tube inserted down the throat); they receive animals back into their care following operations and/or their participation in experiments and they cull animals who are no longer needed. Existing interview-based studies report AT work as challenging (Birke, Aluke, and Michael 2007). Their workplace is heavily regulated and controversial within society at large, which impacts on their day-to-day practices and emotional well-being (see also Davies and Lewis 2010). They are not scientists or budget holders yet they carry “the technician’s burden” (Birke, Aluke, and Michael 2007, 99) acting as “buffers between the scientists and the animals” (Ibid., 103). Indeed, ATs often position themselves as advocates for the animals in their care and correspondingly see a concern for animal welfare as central to what they do.

In this article, we describe in detail the work of ATs and its relation to the guiding ethical principles for humane animal research known as the 3Rs: *reduction* in the number of animals used, *refinement* of housing conditions and experimental procedures to improve animal welfare, and seeking out *replacements* for animals in scientific research. We offer an interpretation of their work as an example of putting animal welfare and ethics into practice and thus deserving of greater attention. Drawing on ethnographic research and interviews conducted across a range of UK universities, we ask *firstly*, how do the 3Rs inform both what is expected of ATs (in terms of the legislation and the oversight of their line managers) and what they expect of themselves? Secondly, to what extent is AT understanding of, and implementation of, animal care work captured by the 3Rs concept? Thirdly, collectively, as a package, how does the work of ATs speak to cross-disciplinary debates about how care can be improved through influencing behavior changes and an emotional engagement with doing the right thing for the “object of care” (Puig de la Bellacasa 2011)? Therefore, we reflect on our findings not only as a contribution to a growing body of work in practical ethics (Smith and Jennings 2009) and critical bioethics (Hedgecoe 2004) but also as a contribution to studies principally found in human health and well-being literature that argue for attention to knowledge practices for improving care (Pols 2014; Mol 2006).

We begin by setting out the broader theoretical context of our work and how it relates to the emerging work on the social science and history of laboratory animal welfare as well as to broader work on the relationship between performative ethics and care. We then turn to outline how laboratory animal welfare is defined and regulated and explore the concurrent professionalization of animal technology. Sections “Responsibility for Implementing the 3Rs: Exploring the Role of ATs” to “Limitations of ATs’ Ability to Respond” explore how the contributions of ATs to the work of refinement and reduction not only meet, but in many cases exceed, the demands currently placed on them by legislation. These contributions, we argue, reflect the need to pay attention not only to how care is conceived but also to how it is practiced within specific contexts or “packages” (Mol 2006). Specifically, we look at the role of ATs in both implementing and innovating refinements (particularly in animal care and husbandry) as well as some of the limitations they face in putting their knowledge of how best to care into practice. Finally, we reflect on the contributions this work can make to current theorizations of the relationships between care, regulation, and technology.

## Care Work and the Social Science of Laboratory Animal Science

Earlier work at the interface of social science and animal research sought to unpack the politics of antivivisectionist movements (see, e.g., Jamison and Lunch 1992), noting that over time these debates seemed to be becoming slightly less polarized (Moss 1984; Sanders and Jasper 1994) and considering the implications for regulation (Zola et al. 1984). Recent work on the social science of laboratory animal welfare has begun to focus more explicitly on the practices of animal research, providing insight into the history (Kirk 2008, 2014), present (Birke, Aluke, and Michael 2007; Holmberg 2008), and future (Davies 2011) of human–animal relations within laboratories. There are also a number of ethnographic studies that examine the human–animal relation in animal experimentation (Johnson 2015; Despret 2015; Nelson 2016). However, there is very little discussion of animal husbandry and an absence of literature that directly addresses the 3Rs in relation to the working lives and practices of laboratory animal technicians.

Here we combine a focus on ATs with a concern with their role in providing care. Care, in Maria Puig de Bellacasa’s words, is simultaneously “a vital affective state, an ethical obligation and a practical labor” (2012, 197). This definition helpfully identifies the multiple forms of care that ATs negotiate within their work: feelings of and for caring, an expectation that they should care for animals, and practical tasks of animal care husbandry such as cleaning, feeding, and so on. Kirk (2014) describes the historical identification of “the stressed animal” in animal welfare science, the consequential development of the 3Rs humane experimental technique (Russell and Burch 1959), and a growing emphasis on “how researchers, animal caretakers and animal technicians formed part of the social environment for laboratory animals” (Kirk 2014, 249). This change in understanding of the animal’s environment is significant to the articulation of care in its multiple forms within the laboratory, yet how that shapes the contemporary day-to-day practices of animal technicians is poorly understood. Within animal studies, including animal welfare and veterinary science, there is considerable expertise for assessing animal welfare against a series of established criteria and indicators. However, within laboratory animal welfare, there is an increasing concern that implementing the principles of the 3Rs involves more than making provisions for and monitoring good welfare outcomes; it also involves the development of a “culture of care.” Below, we explore the adoption of this term as part of wider shifts in the practices of laboratory animal technology. Here we highlight the use of the term to recognize a more complex and nuanced understanding of care as a set of practices, behaviors, and atmospheres that needs to be cultured.

This interest in “culturing care” is echoed by some animal welfarists, who are adopting the language of intervention and behavior change to address this challenge:

Intervention is the term given to a “systematic attempt to change peoples’ behaviours” (Rutter and Quine 2002) and although our goal is to improve animal welfare, the reality is that interventions have to be targeted at the people who hold animals in their care…. The process involves not only passing on knowledge of what needs to be changed (derived from scientific studies and risk factor assessments) but motivating and empowering people to implement changes to their systems, management and daily routines. (Whay 2007, 17)

This impulse contributes to a growing body of work that is interested in reflecting critically upon what leads people to engage in moral practices associated with goods: good care for others as citizens (Pykett et al. 2010), good care for the self (Vogel and Mol 2014), and good practices for the environment (Barr 2014). Rather than a focus on moral education drives, the intent in this work is to understand the forms of practices that support “ways of doing” or “ethical doings” that work toward good things. Much of this literature discusses this in terms of behavior change, by drawing on social practices theory (Shove 2010), but there is a growing literature that draws on theories of practice to emphasize how care is afforded through more-than-social materialities and affect (Roe and Greenhough 2014). The animal house is a place where humans, animals, and technologies are in constant interplay through experimentation and where they must collectively react to regulatory pressures, the principles of the 3Rs, and the changing bodily needs of animals. In this context, we ask how is it possible to improve and deliver good care? Furthermore, rather than focus on how care delivery is improved through isolated human behavior change, we embrace all the various sociomaterial and technical elements that are part of the “package” (Mol 2006) of delivering good care.

Animal technicians, and the relationships they form with the species and individuals they care for, are a key part of this package. Importantly, as Despret (2015) and others have argued, the agency of the nonhuman animal can shape experimental practices and outcomes. Here we argue that it also shapes the delivery of animal care. For Mol, the day-to-day “tinkering” of care work is based on a close and ongoing relationship between caretaker and cared for, and what Haraway (2008) terms a capacity to “share suffering.” However, with the exception of Haraway’s (2008) discussion of the care “responsabilities” of a laboratory animal caretaker, the ethics, and care literature within animal studies tends to focus on domestic animals, for example, Haraway’s (2003) dog. Furthermore, for Mol, this relationship forms only part of the package that shapes the form of care practices. In the animal house, care practices could include (among other things) the genotype of the animals being cared for, the experiment, the local laboratory culture, the technologies used, and the care practitioners. Mol’s approach echoes our conviction that ethics in animal research must be understood not only through the normative, utilitarian values which may (or may not) underpin societal justifications for animal research and the prioritization of a concern for human health, but also through the moral activity of caring-for experimental subjects and how this is shaped with and through the laboratory and animal house environment.

To date, other members of the scientific community who are in traditional positions of power, for example scientists (Hobson-West 2012) or regulators (Schuppli 2011), have dominated science and technology studies research on laboratory animals. We suggest studying ATs offers a novel insight into processes of animal welfare decision-making by virtue of ATs daily proximity to the animals, but one which is rarely seen or discussed due to ATs underrepresentation in decision-making bodies. This is significant because ATs have a very different relationship to the animals in their care. Unlike the rules, regulations, and scientific objectivity that frame how animals are used in the experiment, ATs are given latitude to implement care practices for meeting specific animal or animals’ needs. These practices can happen in an ad hoc fashion and may be work in progress. Therefore, it is relevant to explore the knowledge traditions and practices of animal technicians and how they relate to the articulation, collection, and making available of the techniques for care (Pols 2014). This approach reveals how they pass knowledge and practices among one another, and what informs ethical engagement in particular research establishments and among the wider AT community.

## An Ethnographic Approach

Our evidence is taken from a two-year Wellcome Trust funded research project (2013-2015), exploring how ATs perform ethical as well as emotional labor while caring for laboratory animals. The project draws on a rich combination of participant observation undertaken at two different university animal facilities and a series of three successive interviews undertaken with each of seven (two as a joint interview) junior animal technicians (from a range of UK university facilities) during the first two years of their career (*N* = 18). Participant observation (Laurier 2003) can provide detailed insights into day-to-day practices (e.g., adding environmental enrichment, killing) that are hard to capture in an interview conversation and has proved particularly effective in illuminating human–animal relationships in Scandinavian research on laboratory animal science (e.g., Holmberg 2008). In-depth interviews (Longhurst, 2003, 117) allow social scientists to explore ethics from the “bottom-up” (Hedgecoe 2004), arising from experiences of trying to do the right thing. Repeat interviews (at approximately six monthly intervals) show how junior ATs’ skills, experiences, and responses change over time and allow the researchers to build the rapport and trust necessary to tackle challenging issues. We also undertook further in-depth interviews with key stakeholders in laboratory animal welfare including animal facility directors, members of the Institute of Animal Technology (IAT) and Animals in Science Education Trust, and relevant nongovernmental organizations (*N* = 9) as well as further participant observation and informal conversations during tours of facilities and professional meetings and conferences. These additional interviews and observations help elucidate the broader context within which the ATs we were focusing on worked and provided alternative perspectives on some of the issues and experiences they discuss. It should be noted that this research focused exclusively on the UK, and given the significant international differences in the regulation and practice of animal research, it would be hard to generalize from these data to non-UK contexts. Interviews were transcribed verbatim and interview and fieldwork diaries were thematically coded using N-Vivo 10.2.0 software to identify key emergent themes and issues. To preserve interviewee confidentiality, all names have been replaced by pseudonyms and a broad indication of interviewee’s position in the lab (e.g., junior AT).

## The Principles and Professionalization of Humane Animal Experimentation

The “3Rs” were first proposed by Russell and Burch (1959) in *The Principles of Humane Experimental Technique*. In the wake of increasingly vocal antivivisectionist protest, and growing concerns about animal welfare, they have evolved to become central to the governance of animal research, formalized in regulations including the UK Animals (Scientific Procedures) Act (ASPA, 1986, revised 2012) and more recently the European Directive 2010/63/EU on the protection of animals used for scientific purposes. In this article, we focus on refinement and to a lesser extent reduction, as these are the two areas identified by our participants where ATs may play a key role. The development and, more significantly, the validation of replacements for animal models require levels of expertise and status in the scientific community that would make it difficult for ATs to engage.

**Figure 1.**
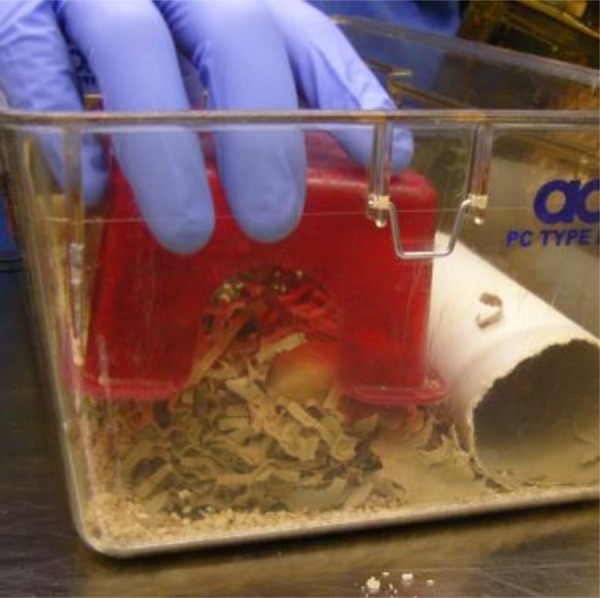
Installing environmental enrichment (red plastic house, nesting material, and chewable “fun tunnel”) in a mouse cage.

Refinement specifically focuses on “improvements to procedures and husbandry that minimize actual or potential pain, suffering, distress or lasting harm and/or improve animal welfare in situations where the use of animals is unavoidable” (Medical Research Council 2014, 4). Russell and Burch (1959) distinguish between two directions for refining animal experiments. The first, “*contingent inhumanity*,” refers to the pain and distress caused simply by rearing and housing animals in a laboratory environment and incorporates the animal’s entire life course, from breeding to transport, housing, handling, health, and death. AT’s practice directly informs the rearing and housing of animals in a laboratory environment, showing interest in providing environmental enrichments, including bedding, chew toys, and shelters (Figure 1). Although they may not control budgets for the style of housing, or be in charge of requests for more animals to be bred, AT’s practice can enrich the animal’s experience of living in a laboratory and some of the conditions of this experience are in the remit of ATs decision-making or advocacy. The second form of refinement, “*direct inhumanity,*” refers to the pain, suffering, and lasting harm caused by the experimental procedures themselves. Some ATs are involved in carrying out experimental procedures that require careful skills to execute them well, for example, extracting and injecting, oral gavaging, applying modes of restraint, or administering anesthetics and analgesics.

Reduction is defined as

the principle that, wherever a programme of work involving the use of protected animals is carried out, the number of protected animals used must be reduced to a minimum without compromising the objectives of the programme; […] The principle of reduction should apply to methods of breeding protected animals as well as their use in procedures. (Home Office 2014a, 27)

This is principally achieved through the process of experimental design and (in a UK context) often refined during the process of applying for a project license through discussions between the principal investigator (lead researcher) and Home Office Inspector (Fieldwork notes, June 25, 2015). However, as we shall outline below, some ATs suggest colony management (i.e., the numbers of animals bred and culled) could be made more efficient (with less wastage) by making greater use of AT knowledge and expertise.

The adoption of the 3Rs has been accompanied by a professionalization of animal care in the UK, some areas of Western Europe, especially Scandinavia, and to an extent in the United States and beyond through the operations of Association for the Assessment and Accreditation of Laboratory Animal Care international. Taking the case of the UK as our particular example, in the decades leading up to ASPA laboratory animal husbandry was usually performed by care-taking staff who had very little formal training. In-depth interviews (on which more below) with animal technology professionals suggest that many of these workers came from agricultural backgrounds and that the work was predominantly male-dominated. Similarly, pre-ASPA Home Office Inspectors had largely been drawn from the military; post-ASPA, there was an influx of vets in the role who emphasized refinements such as analgesics (Interview with IAT trainer, 30 August 2012). However, our interviews with animal facility directors also suggest that the kinds of people entering the profession have changed over the last thirty years. Although people come with a wide range of backgrounds, from school leavers to university graduates, most are women and most have some form of qualification in animal care and welfare and considerable experience in animal husbandry. Of the seven junior ATs we interviewed (all under thirty), six were female and five had some experience of higher education relating to animal care, two at graduate and one at postgraduate level.

Correspondingly, over the past thirty years (since the implementation of ASPA), the training of animal care staff, now relabeled as ATs, has become increasingly standardized and largely overseen by the IAT (Interview with IAT trainer, 30 August 2012). ATs follow a series of college courses and modules during their first few years of employment, and beyond if they have the interest and motivation, where they learn aspects of animal husbandry, physiology, behavior and biology, methods of humane killing, ethics, the principles of the 3Rs and legislation and regulatory compliance. All our junior ATs spoke about attending college either as in-house training or through day-release schemes and about working toward getting their personal license (on which more below). For many, especially those with some background in higher education, the availability of a clear career path was an appealing aspect of the job. Within the professional world of Laboratory Animal Technology, this training is seen as key to teaching ATs to meet the regulatory requirements of ASPA and to implementing the 3Rs. However, while recognizing the importance of formal training programs and regulation in ensuring laboratory animal welfare, in the next sections, we explore how ASPA, Home Office guidelines, and IAT training programs define the responsibilities for ATs in implementing refinement in very particular ways. We argue that these ways do not entirely capture the commitment to implementing and innovating refinements that we observed during our research with junior ATs or growing concerns over the need for further reduction in the number of animals used.

## Responsibility for Implementing the 3Rs: Exploring the Role of ATs

Before research using laboratory animals can be carried out, there must be in place (i) an *establishment license* for the location where the work is to be conducted, (ii) a *project license* for the specific program of work (up to five years), and (iii) a *personal license* for each individual carrying out procedures on animals, including any methods of euthanasia not permitted under schedule 1^2^ of ASPA. Personal license holders, including many ATs, must “act at all times in a manner that is consistent with the principles of replacement, reduction and refinement” and “not allow an animal to experience severe pain, suffering or distress that is likely to be long-lasting and cannot be ameliorated” (Home Office 2014b, 14). Recognizing that this edict is open to broad interpretation, the Home Office provides a list of specific responsibilities for personal license holders (Table 1), some of which (e.g., filling food hoppers or water bottles with previously dosed diets or liquids) may be delegated to nonlicense holders (Home Office 2014b, 37).

**Table 1.** Home office guidance on the specific responsibilities of Home Office Personal License Holders, adapted from Home Office 2014b pages 35-36.

Responsibilities of Personal License Holders: being responsible for the welfare of the animals you have performed procedures on and ensuring that they are properly monitored and cared for; knowing the techniques and species involved, what the consequences of performing procedures on them will be and the signs of pain, suffering, distress or lasting harm in that species; taking precautions to prevent, or reduce to a minimum consistent with the purposes of the procedure, any pain, suffering, distress, or discomfort to the animal which may or may not lead to lasting harm. This may include using medication where appropriate, such as sedatives, tranquillisers, analgesics or anaesthetics, as well as other appropriate methods such as husbandry measures, which increase animal comfort or improve access to food and water; telling the project licence holder immediately if you think that the severity limit of any procedure, or other limitations (constraints) upon adverse effects, have been or are likely to be exceeded and agreed humane end- points have not or cannot be applied; getting and following veterinary advice and treatment, where needed; arranging for the care and welfare of an animal when you are away; making sure that any animal that is in severe pain or severe distress, which cannot be alleviated, is painlessly killed using an appropriate method; ensuring that neuromuscular blocking agents (if authorised to be used) are used in combination with anaesthesia and analgesia as required by the project licence; ensuring animals are killed by an appropriate method at the end of the procedures if the animal is suffering or is likely to suffer adverse effects.

Similarly, the Home Office (2014a, 3) *Code of Practice for the Housing and Care of Animals Bred, Supplied or Used for Scientific Purposes* “encourages licensed establishments to continually improve their standards of care and accommodation in line with the principles of the 3Rs (replacement, reduction and refinement).” In addition to the stated “minimum legal requirements” (Introduction and Care Work and the Social Science of Laboratory Animal Science sections), the guidelines go further. An Advice section “seeks to encourage establishments to promulgate high quality animal welfare and high quality science, which may go beyond the minimum requirements, where applicable” (Ibid., 7) and talks about the need for establishments to strive to “adopt higher standards where practicable and applicable” (Home office 2014a, 3).^3^ This somewhat ambiguous classification of guidelines into “legal requirements” and “additional advice” makes space for ATs (among others) to not only meet minimum requirements but to strive to exceed them, challenging Mol, Moser, and Pols’s (2010, 7) suggestion that rules and regulations risk eroding practices of care. However, this challenge will only succeed if those with the responsibility for implementing these refinements have both the motivation and the means to strive.

## Implementing Refinement through Following Protocol

The Home Office license system and guidance are very visible in the discourse of ATs, who all demonstrated a keen awareness of the importance of compliance with the conditions of project licenses. In the course of our two years following junior ATs, many of them went through the process of applying for and obtaining their personal license. All were equally aware that the penalties for infringements are severe, citing the example of those involved in a recent infringement at Imperial College London, who were sanctioned, received letters of reprimand, and were required to undertake further training (Home Office 2014c). Interestingly, the subsequent independent report commissioned by Imperial College––the “Brown report” (Brown 2013)––saw training as the key issue and recommended the formal designation of a Named Training and Competency Officer, alongside the Named Animal Welfare and Quality Officer to mentor and monitor staff and help sustain a culture of care.

Within the animal research facilities we visited, considerable emphasis was placed on cultivating a culture of care, a phrase that for managers seems to express a commitment to high professional standards and to building relationships of trust and confidence among staff at junior and senior levels. Cultivating a particular workplace atmosphere is seen as key to encouraging junior staff to speak out when they have concerns about any animal in their care:

But I always tell the staff that if there’s any [welfare] problem, they come and see me. If I’m the problem, then they have to take that further and contact my boss…. (Interview with Facility Manager, 5 September 2013)

For Mol, care is often the antithesis of both scientific objectivity and rules and regulations. Drawing on the enlightenment tradition, Mol, Moser, and Pols (2010, 7) suggest that “[t]o the sciences, bodies were interesting in as far as they could be objectified and explained in the laboratory, but not as they shuffled about, gasped for breath, gobbled up or lingered over food, talked, screamed, or needed to be soothed.” Echoes of this perspective can be heard in AT’s conviction that their role is to challenge researcher’s tendencies to remain insensitive to animal suffering:

Everything we do is governed by the Home Office…all the experimental work being done is under a licence. And that licence has limits…what we get trained for, is to look for these signs and if [we] see it’s exceeding the licence, then report it quickly so that animal doesn’t suffer. So that’s an animal technician’s job, to police the researchers, the scientists. (Interview with Mark, Senior AT, 5 September 2013)

While we might question the extent to which all researchers are insensitive to animal pain,^4^ both the Home Office guidelines, and more broadly the training programs and culture of care cultivated within animal research facilities, serve to define a role for the ATs focused on the implementation of experimental procedures and protocols in accordance with the project license, on monitoring of welfare outcomes and severity limits, and on raising concerns if they feel animals are suffering beyond was has been permitted under license. However, below, we will suggest ATs draw on other skills that might also be seen as key to refinement.

## ATs Innovating Refinements

Beyond following experimental protocols, many ATs address the needs of animals in their care using their intuition or, in other words, they work to refine animal’s experiences of contingent and direct inhumanity. Many of these forms of refinement become available through their developed somatic sensibilities (Greenhough and Roe 2011) derived from a combination of their training, an often lifelong interest in animals and long periods of working with a particular species. In these examples, ATs discuss different forms of environmental enrichment they use with mice:

You give them toys, […] a nest to play with and I like to put in some cubes as their food ‘cause then they get a chance to pick that up and play with it…. And another good thing to do is put tissue where their water bottle is because they will—, they like to, sort of like, pull off pieces of that and turn it into nest. (Interview with Claire, Junior AT, 18 November 2013)

I’ve noticed that if [a certain strain are] by themselves, they constantly flip on their cages, flip and flip and flip and flip. So we’ve got like little igloo houses that we put in there and it’ll stop them. (Interview with Debbie and Fiona, Junior ATs, 15 November 2013)

These are subtle refinements, changes not to the experiment itself, but tinkerings to improve how these unique animal species strains (often poorly understood by anyone except their nominated AT and a researcher) can live better within the animal house. Claire understands her action as a “good thing to do,” Debbie and Fiona understand them as enrichments to avoid a behavior. ATs articulate their responsibility for addressing these aspects of laboratory mouse lives and by doing so enact the responsibility imposed by the Home Office. However, it also illustrates Mol, Moser, and Pols’s (2010) argument that abstract ethical principles rapidly become synonymous with care practices as soon as ethics are studied in practice. Rather than making recourse to universal values or norms (such as those which argue for or against vivisection), the focus becomes on ethical-doings or the specific local negotiations of performing laboratory animal care.

**Figure 2.**
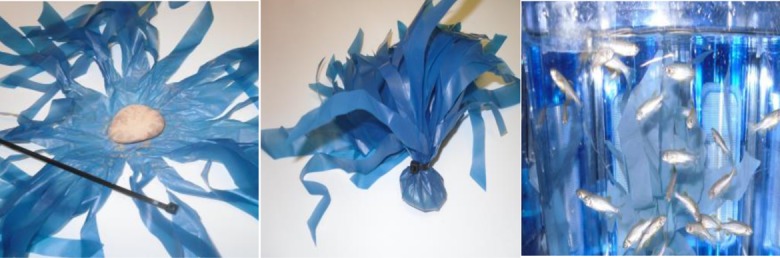
Making weeds to enrich zebra-fish tanks.

Laboratory animal care is facilitated by ATs directly observing and sensing animal’s needs and through the course of their work leads to them developing innovative refinements. Here the work of ATs further illustrates both “practical tinkering” and “attentive experimentation” (Mol, Moser, and Pols 2010, 13) as good and care-full things to do. At a recent Institute of Animal Technology (IAT) Congress (2015) meeting, we viewed numerous posters from ATs focused almost entirely on refinements to reduce animal stress and suffering and improve welfare, from the specific care needs of pruritic (to have itchy skin/hair loss) and aging mice (Carpenter 2015) to the replacement of telemetric cages with hydrophobic sand as a means of collecting urine samples (Smith 2015). Similarly, in our ethnographic work, we noted how individual facilities have developed specific local-level refinements used to reduce harm and suffering during experimental procedures. For example, one unit used Vaseline on mouse tails during tail bleeds to stop “the hairs on their tails getting ripped out” and going “really sore and horrible” (Interview with Claire, Junior AT, 5 August 2014). Another unit had developed a novel delivery method for analgesics in mice postsurgery using strawberry-flavored jelly (Fieldwork notes, 2015). A different group of ATs at a fish facility had developed a method for making their own “weeds” from plastic garden bags, pebbles, and twine (Figure 2), an innovation which generated considerable extra work as the weeds had to be bleached over a period of months so they did not release hormones into the water, as well as making tanks more time-consuming to clean.

Striking is that beyond the immediate environment, and perhaps presentations at IAT Congress or in professional journals such as *Laboratory Animals*, refinements innovated by the AT community appear to not travel in the same way as refinements that come from laboratory animal welfare science itself (see Weary 2013). In other words, refinements come in different forms, and there is not a formalized culture of publicizing AT innovations in this field beyond the local AT community except in an ad hoc manner. There is some evidence to suggest that Home Office Inspectors––who move between sites––may share their insights from other sites. More senior staff may share innovations and conventions at professional meetings or at workshops for ATs run by the National Committee for the 3Rs (who also host website for sharing best practice) or the Royal Society for the Protection of Animals. We also were told of a Listserv for the AT community that could be used more for the dissemination of new refinements. However, the existing repertoire of refinements so painstakingly developed by ATs are unlikely to ever feature in the discussions of ethical review boards. Nor is the AT cost, in terms of time and labor to develop and further their repertoire, found in funding applications that support animal research.

## ATs’ Experiences of Reduction

We get some researchers who just breed and breed and breed, and then go, “Oh, my bill’s too high,” and then just kill a lot of them. And it’s like, well why breed them in the first place?…I like the fact that some of the researchers […] only breed what they need. I wish more of them were like that. (Interview with Eleanor, Junior AT, 21 August 2013)

We found ATs critical at times about breeding and culling practices they were asked to carry out, and their insights may have implications for implementing the 3Rs principle of reduction. They spoke often about the stress they experienced killing healthy animals:

I found it quite stressful really in lots of ways, especially when it came to culling more healthier animals because, like, in my head it’s kind of like, you know they’re healthy, why do we have to kill them? (Interview with Claire, Junior AT, 18 November 2013, talking about mice)

When animals become excess to experimental requirements, they are culled. The reasons may include extended holiday periods or researchers struggling to afford to keep the colony going or because the experiment only requires some of a litter (e.g., those of a certain gender or genotype). This was a key issue for many of the ATs we spoke to, some of whom suggested that if researchers had to deal with practical and emotional consequences of overbreeding (i.e., culling excess “stock”) they would be more careful to only breed what was needed. Some ATs recognized that the unpredictability of breeding processes makes it impossible to guarantee the number born in a litter with the desired genetic characteristics, but in comments about this practice, ATs emphasize the health of the animals and their frustrations about these culls.

None of the ATs we spoke with raised the idea that life in the laboratory may in fact not be one worth living, which is an increasingly popular discussion point in animal welfare (Weary 2013). Rather, the focus of their discomfort with killing healthy animals tells us something about the positive culture among the AT community, how welfare is defined in AT training, and the belief that for laboratory animals life outside of the experiment, if they are healthy, is good. However, arguably there is no simple concept of good health or quality of life for strains of mice selectively bred as models for human disease. Thus, what comes across strongly from ATs’ comments is the value they place on care to support life as opposed to valuing their capacity to end life.

As Birke, Aluke, and Michael (2007) also found, the acts of killing ATs carry out are easier if the animals have contributed to research. It is not as simple as saying euthanasia is not something that ATs have no understanding of, for they routinely euthanize individual animals that are in obvious discomfort. Instead, it is killing groups of animals that are not in pain and appear healthy (yet living within laboratory conditions and that potentially could endure suffering through involvement in an experiment) that frustrates them. In other words, euthanasia is held as a “good thing” in some circumstances and not others. Despite the strong feelings among the AT community about the practice of culling, current Home Office guidelines make no reference to the role of ATs in colony management––a role usually assigned to the project license holder. AT responsibilities are confined to the care and husbandry of breeding stock. However, some ATs actively sought to work with researchers to limit what they perceived to be overbreeding, bringing their knowledge of breeding particular strains and the current state of the breeding colony into conversation with researchers making requests for animals.

It’s the researchers, they will say, we want so many of this strain…[to]…get the numbers that they want. But quite often or not, we’re very good at saying, we don’t [need] anymore. (Interview with Claire, Junior AT, 18 November 2013)

One facility even offered certificates to researchers who successfully managed their colonies with minimum waste. Furthermore, most of the ATs we spoke to felt that refinement could be further improved if more control over breeding and colony management was passed to ATs, alongside the use of new computer technology to improve colony management and tracking:

I think, the bigger problem with breeding is […] the researcher going to the technician and saying, I want this or we can slow down on that now…because we’re done with that for now but we really want to keep that strain going and we’ll have some of this strain going up […] I think we’re looking at getting a new computer system at some point so hopefully that’s going to help with the communication of breeding and stuff like that a bit better. (Interview with Claire, Junior AT, 18 November 2013)

As we have heard, given that ATs often bear the practical (and arguably emotional) labor of culling excess stock, it could be more explicitly recognized that they are an important voice in colony management even though this is not formally recognized in how their role is defined. Euthanasia in the laboratory could also be discussed as a practice related to the principle of reduction. This form of AT involvement may influence local breeding cultures.

## Limitations of ATs Ability to Respond

Having explored an expanded role for ATs in delivering refinement and reduction, in this final section, we want to consider three ways in which ATs’ capacity to exceed the minimum requirements of ASPA and the 3Rs might be limited. Firstly, while ATs are encouraged to speak out, many of those at junior levels still face challenges in doing so:

There are certain things that you see that you want to change and at the minute it’s kind of like I want to be able to change it **but I also don’t want to step onto anyone’s feet**…I find that quite challenging to find where the line is. (Interview with Carrie, Junior AT, 18 November 2013)

This sense of being unable to speak is reflected more broadly in the relative underrepresentation of junior ATs on ethical review boards. In a recent poll conducted at IAT Congress as part of a workshop training and encouraging ATs to join their local AWERB, Hawkins, Farmer, and Woodley (2015) found that while 82 percent of participants would like to sit on their local AWERB, only 34 percent did so; of those who did not the biggest reason (27 percent of respondents) given was that “I didn’t think someone with my role could be a member.” *Therefore, we might argue it is not just rules and regulations that place constraints on the practice of care but also the institutional environments within which care is situated and the power dynamics at work at those sites, which shape how rules and regulations are (or are not) implemented.* In practical terms, this leads to the imperative for more to be done to address the sense of powerlessness many ATs feel to act on their impulse to care and thereby make their jobs both more interesting and rewarding. Workshops, such as those run at the IAT, combined with institutional policies that actively promote the inclusion of ATs on ethical review boards and similar and opportunities for dialogue between ATs and researchers, could play a key role in altering the balance of power.

Secondly, as AT refinements are not developed through the scientific method, they are seen as being of questionable use and value. Refinement is perhaps the area of welfare where most scientific research has been directed (see, e.g., Flecknell 2002). This work has made significant contributions to improving animal welfare. However, it is unclear where the refinements developed by ATs fit into this process. Flecknell (2002, 74) suggests that of all the 3Rs, refinement is the most problematic:

Much of our judgment of what represents Refinement is based on little more than common sense. We make assumptions about animals and their feelings that often have little scientific basis. In many instances we may be correct, but these assumptions may become incorporated into institutional or national policies, without any attempt to verify them.

Furthermore, the lack of scientific evidence as to the impact of refinements also raises concerns among some researchers and the scientific community that they could introduce unwelcome variables into experiments. As Flecknell (2002, 74) notes, “enrichment strategies may change the biological characteristics of laboratory animals, and may also increase the variability of some parameters.” This may call into question the comparability of results derived from experiments using animal models subject to enrichment, on one hand, with those conducted with models who had not had enrichment introduced, on the other. A tension therefore emerges between the often-repeated rhetoric that “good welfare is necessary for good science,” and the need to be able to compare the results of experiments that are conducted in contemporary welfare-enriched environments with those undertaken under past welfare and husbandry regimes. It is perhaps at this point that care finds itself once more in conflict with scientific objectivity.

Finally, scale also may limit ATs’ role in animal care. As Davies (2012) notes, the kinds of attunement and care shown by some of the ATs cited above requires time that is often unavailable in large-scale mouse breeding units. As facilities expand and the number of animals increases, finding time to spend with each animal, let alone to innovate refinements, becomes increasingly challenging. Indeed, the increasing use of technology, particularly in more industrial breeding and testing establishments, can serve to prevent ATs from developing the skills and experiences needed to perform care work:

And as you look at the equipment that’s come in on the line over the last few years, individually ventilating cage systems, animal technicians are slowly becoming isolated from the animal as the systems take over. And if you look at some of the accredited breeding establishments, they don’t even clean the animals out. You know, some of them have got systems whereby the cages are suspended over trays, and so the faeces and urine drops through a grid floor and the tray is then just sluiced away. So animal technicians are not picking them up and scraping boxes out […] They’re breeding tens of thousands, hundreds of thousands of animals possibly per annum, and they never, ever know the animals that they’ve bred, what they’re going to be used for. So how can they actually have sort of a strong identity, how can they actually feel for what they’re doing? (Interview with David, Facility Manager, 15 May 2012)

New efficient automated systems may in some senses be seen as a form of care, providing more hygienic and disease-free environments for both humans and animals and reducing both ATs’ manual labor and the stresses animals may experience from being handled. However, these technologies also serve to alienate ATs from their animal subjects, thereby limiting the interactive, attentive encounters that we have suggested may be a key to ATs developing the skills and sensitivities they need to innovate refinements to husbandry and colony management.

## Conclusions

This article builds on recent work in Science and Technology Studies that demonstrates how care is relational, performative, and multiple (Mol 2008; Mol, Moser, and Pols 2010; Puig de la Bellacasa 2012) and argues that ethics in animal research needs to be understood not only through normative, utilitarian values but also through the moral activity of caring-for experimental subjects and how this is shaped with and through the laboratory and animal houses’ regulatory, technical, and affective environments. Care is often seen in opposition to scientific objectivity and protocols that effectively somatasize experimental subjects, reducing them to bodies to be observed and tested (Mol, Moser, and Pols 2010). However, regulatory and management bodies’ calls for the promotion of a culture of care within animal technology practices suggest a recognition of care as a relational practice that can be fostered or hindered and that caring for experimental bodies is valued by the scientific community and is not in opposition to scientific objectivity. In closing, we wish to emphasize how, in turn, studying the care work of laboratory ATs complements and extends existing understanding of the relationships among care, regulation, behavior, and technology.

Firstly, we noted how, in contrast to Mol, Moser, and Pols (2010, 7, see also Singleton 2010), suggestion that rules and regulations risk eroding practices of care, *regulation in the case of laboratory animal welfare places ATs in a role with both a mandate to care for their subjects, and, in the case of existing Home Office Code of Practice* (2014a, 3), *some degree of latitude as to how they do so*. Additionally, ATs’ ongoing commitment to “staying with the trouble” (Haraway 2016), seeking not only to implement but also to innovate refinements, to “tinker” (Mol 2008) to improve animal care practices and thus the welfare status of the animals in the course of their day-to-day care work sits comfortably within the 3R principles of refinement and reduction. Mol (2008) suggests that the tinkering undertaken in the pursuit of care is underpinned by a moral imperative. In other words, ATs are “tinkering with care in the persistent hope of improving it” (Ibid., 413). These are virtues upheld by the 3R principles. From this perspective, what is important about the 3Rs is not only the principles they advocate, but also the extent to which those principles can be used to evoke cultural and behavioral changes that lead to day-to-day improvements in the practice and provision of care.

Because “values” intertwine with “facts,” and caring itself is a moral activity, there is no such thing as an (argumentative) ethics that can be disentangled from (practical) doctoring. **You do what you can while watching out for the problems that emerge—in bodies or in daily lives, caused by the disease or its treatment**. (Mol 2008, 79)

Secondly, we explored how ATs sought to tinker with the various socio-material and technical elements that are part of the “package” (Mol 2006) of delivering good care, showing how care is not only responsive, relational, and multiple but also innovative and creative––for example, the making of artificial weeds for zebra-fish colonies (Figure 2). *How* ATs both implement and innovate refinement and reflect upon imperatives for reduction, constitutes the ongoing “package” (Mol 2006) of care that emerges within animal laboratories––something that regulators and managers are arguably trying to capture by supplementing a focus on the 3Rs with increasing references to the need for a culture of care. As Mol and her colleagues (Ibid.) also remind us, care is not just an object to be achieved but also “a mode, a style, a way of working.” Here developments in animal technology mirror those in other (we would argue) allied professions, notably nursing, which has also seen a growing emphasis on the need to promote a culture of care (Rafferty et al. 2015) as well as work in animal welfare that *emphasizes the role of behavior change in improving animal care* (Whay 2007). Indeed, the growing use of the term “culture” within regulatory codes such as those studied here is one of many attempts to capture something of the uncodifiable dimensions of care work. We further suggest, contra Mol, Moser, and Pols’s (2010, 7) suggestion that rules and regulations risk eroding practices of care, that regulation can therefore play a positive role within the practice of care, opening spaces for (in our example) ATs to innovate toward better animal welfare.

Thirdly, in contrast to her more negative view of regulations, Mol (2008) has also suggested that technology can enable the practice of good care. We found that this may happen through unlikely processes. Where Singleton’s (2010) analysis of the UK’s computerized Cattle Tracing System described a regulated, record-keeping technology in tension with the provision of good care, the ATs we studied saw no tension between the introduction of record-keeping technology and the level of their care. The prospect of a computerized tracing system, the ATs indicated, would allow them to better monitor and convey information about the breeding of animals within their unit, which in turn would give them better control over animal reproduction rates leading to improved care and reducing overbreeding and needless deaths.

Technologies, Mol argues, have indeterminate effects and can shift the practical and moral frameworks of existence, sometimes in ways that allow better care outcomes to emerge. This was the hope of Russell and Burch (1959, unpag.), who suggested that one of the implications of increasing automation within the animal technology field is that it would give ATs more time with animals:

Relieved of routine chores, the animal technician is free for more interesting and stimulating work. He or she is able to take a much greater and more informed interest in the health and behavior of the animals and the progress of the investigations. In particular, such a technician has time to take a personal interest in, and devote personal attention to, even individual animals. The whole human–animal relationship becomes less impersonal, a very important factor in a large animal house. Much may, therefore, be hoped from automation, both in raising the status of the animal technician and in improving the lot of his or her charges.

We share Russell and Burch’s (1959, unpag.) emphasis on the quality of the AT–animal relationship as being key to refinement. They imply that giving the ATs more time to develop relationships with the animals could play a key role in “raising the status of the animal technician and in improving the lot of his or her charges.” However, we would challenge the straightforward assessment that automation will facilitate greater interaction between ATs and the animals in their care. As Holloway (2007) has also shown, automated systems in animal care have a significant impact on human–animal relations, including offering greater independence for the animal to be less reliant on a human to meet some of their care needs (in Holloway’s case robot milking machines allow cows to be milked as many times as a day as they want and at a time they want). Technology can facilitate independence for the animal and it can free up time for the AT to spend greater quality time with the animal, but there is also the risk that automation limits the interactive, attentive encounters we have suggested above may be a key to ATs developing the relationships, skills, and sensitivities they need to innovate the kinds of refinements to husbandry and colony management discussed above. At worse, it can completely alienate animal and human from each other (see also Niemi and Davies 2016). Thus, it is a more complicated, nuanced picture that emerges. We might ask to what extent automation is having a similar effect in other related care settings, placing at risk intuitive and empathetic relations and the knowledge and insights they produce in the name of improved efficiency. Key here perhaps is the question of what exactly is being automated: is the machine performing a function that might once have been performed by an ailing body (as in the case of Willems’s [2010] study of artificial ventilation and oxygen provision technologies) or is the machine replacing a task that once might have been seen as an aspect of care, such as the process of milking a cow (see Holloway 2007), or is the machine replacing the work of an AT to an extent that substitutes animal-human encounter entirely or considerably reduces time for this relationship to flourish? *In short, a key question may be whether technology is supplementary to the provision of care (such as the computerized breeding records welcomed by ATs) or intended to supplant it (as in the case of fully automated cages)*.

To conclude, we would emphasize the need to understand care as performed in relation to a specific assemblage of regulation, technology, and behavior. Of particular interest throughout this article is how the 3Rs as ethical techniques have shaped care as an ethical obligation that in turn has led to particular laboring of care to take place in the laboratory environment. In Kirk’s (2014) wide-ranging article that discusses the historical development of concern for the “stressed animals” initially in laboratory research and then more latterly intensive agriculture, some interesting comparisons and differences are articulated across different spaces of animal care. For example, the cultures, technologies, economies of laboratory animal research and the rationalities for livestock farming are different. They in turn result in different ways of managing and caring for animals, despite broadly similar approaches for assessing animal welfare across different spaces (Leach, Thornton, and Main 2008). Studying the specific combination of guidelines, enabling regulation and workplace culture and environments that facilitate and encourage ATs to “stay with the trouble” (Haraway 2016) and constantly question the “ethics” of what they do, offers insights about how Mol’s understanding of care as “tinkering” might be used to examine and interrogate animal welfare practices, while also highlighting the limits to tinkering within the laboratory animal environment.

## Notes

1. Across work on laboratory animal science, a range of terminology is used to describe those who perform husbandry, breeding, and routine procedural work within animal facilities including “animal care takers,” “animal research technicians,” and “animal technicians” among others. Use also varies between international contexts (e.g., UK and United States). For example, the United States makes a distinction between animal technicians (who perform husbandry work) and animal technologists (ATs, who are trained to carry out certain experimental procedures on animals). Here, we use ATs to cover both these aspects as this is the preferred term of the Institute of Animal Technology, the professional body for UK ATs.

2. Schedule 1 of the UK Animals (Scientific Procedures) Act (ASPA) refers to methods of humane killing for species protected under UK ASPA which are deemed appropriate to be carried out by any suitably trained individual and which do not require a personal license. These are the first methods junior ATs learn. They are species-specific, but for rats and mice (the most commonly used experimental animals), they include exposure to increasing concentrations of CO_2_ gas and cervical dislocation (breaking the neck), the methods we most commonly witnessed during our research. Use of any schedule one method must be followed by confirmation of death by a second method. For example, following the use of the CO_2_ chamber, an AT may then use cervical dislocation on the bodies as confirmation of death.

3. We are grateful to Gail Davies for pointing us toward this document.

4. Our fieldwork notes and observation suggest that while ATs do sometimes criticize researchers for being indifferent to the needs and lives of laboratory animals, they often also show an admiration for researchers who actively engage with both the ATs and the animals in their care. For example, one AT described the care and attention a researcher gave to zebra fish following surgery. Another spoke of primate researchers being visibly upset at the culling of a subject at the end of a long (over ten years) period of working with that animal.
